# Employing machine learning using ferroptosis-related genes to construct a prognosis model for patients with osteosarcoma

**DOI:** 10.3389/fgene.2023.1099272

**Published:** 2023-01-17

**Authors:** Hui Huang, Zhifang Ye, Zhengzhao Li, Bo Wang, Ke Li, Kai Zhou, Huiyuan Cao, Jiaxuan Zheng, Guangji Wang

**Affiliations:** ^1^ Department of Sports Medicine, Hainan General Hospital (Hainan Affiliated Hospital of Hainan Medical University), Haikou, Hainan, China; ^2^ Department of Emergency Surgery, Hainan General Hospital (Hainan Affiliated Hospital of Hainan Medical University), Haikou, Hainan, China; ^3^ Department of Pathology, Hainan General Hospital (Hainan Affiliated Hospital of Hainan Medical University), Haikou, Hainan, China

**Keywords:** machine learning, osteosarcoma, ferroptosis, prognostic model, tumor microenvironment

## Abstract

Identifying effective biomarkers in osteosarcoma (OS) is important for predicting prognosis. We investigated the prognostic value of ferroptosis-related genes (FRGs) in OS. Transcriptome and clinical data were obtained from The Cancer Genome Atlas and Gene Expression Omnibus. FRGs were obtained from the ferroptosis database. Univariate COX regression and LASSO regression screening were performed and an FRG-based prognostic model was constructed, which was validated using the Gene Expression Omnibus cohort. The predictive power of the model was assessed *via* a subgroup analysis. A nomogram was constructed using clinical markers with independent prognostic significance and risk score results. The CIBERSORT algorithm was used to detect the correlation between prognostic genes and 22 tumor-infiltrating lymphocytes. The expression of prognostic genes in erastin-treated OS cell lines was verified *via* real-time PCR. Six prognostic FRGs (*ACSL5, ATF4, CBS, CDO1, SCD,* and *SLC3A2*) were obtained and used to construct the risk prognosis model. Subjects were divided into high- and low-risk groups. Prognosis was worse in the high-risk group, and the model had satisfactory prediction performance for patients younger than 18 years, males, females, and those with non-metastatic disease. Univariate COX regression analysis showed that metastasis and risk score were independent risk factors for patients with OS. Nomogram was built on independent prognostic factors with superior predictive power and patient benefit. There was a significant correlation between prognostic genes and tumor immunity. Six prognostic genes were differentially expressed in ferroptosis inducer-treated OS cell lines. The identified prognostic genes can regulate tumor growth and progression by affecting the tumor microenvironment.

## 1 Introduction

Osteosarcoma (OS) is a highly aggressive malignant bone tumor that occurs mostly in children and adolescents. The global annual incidence rate is 480 cases per million (Bao et al., 2019; Lancia et al., 2019; Wang S. et al., 2019; Zhou et al., 2020). OS is thought to originate from primitive mesenchymal osteoblasts, typically found in rapidly growing bones, approaching the feet or hands (Mutsaers and Walkley, 2014; Leichter et al., 2017; Gambera et al., 2018). The modern multimodal treatment combining chemotherapy, surgery, and radiation therapy can improve 5-year survival by 60%–70%. However, 40%–50% of patients will develop refractory metastases with poor prognosis (Prabhu et al., 2014; Wu et al., 2015). Therefore, There is an urgent need to improve the treatment and prognosis is a specific cell death pathway caused by iron-dependent lipid peroxides, which may cause the deposition of reactive oxygen species (ROS) (Verma et al., 2020; Wu et al., 2020). Previous studies suggest that elevated ROS concentrations can promote tumor cell growth, but excessive accumulation of ROS may cause irreversible oxidative damage, which, in turn, leads to ferroptosis (Galadari et al., 2017; Hedrick et al., 2019; Yee et al., 2020). Ferroptosis plays important roles in cancer, cardiovascular system, and nervous system diseases (Li et al., 2020). OS is also intimately associated with ferroptosis and involves many potential mechanisms and therapeutic applications (Zhao et al., 2021). Chen et al. (2021) found that KDM4A depletion significantly inhibits *in vivo* OS progression and lung metastasis, and KDM4A knockdown promotes ferroptosis in OS cells, which is a non-apoptotic cell death mechanism. Lin et al. (2021) found that EF24 upregulates HMOX1 to inhibit GPX4 expression and induces ferroptosis in OS cells by increasing MDA, ROS, and intracellular iron ion levels.

The development of high-throughput sequencing technologies continues to accelerate the exploration of cancer prognostic models based on sequencing results. Liu et al. (2021) successfully identified ferroptosis-related gene (FRG) signatures with important prognostic value for bladder cancer, providing a new research direction for future bladder cancer targeted therapy. Li et al. (2021) created features and nomograms of 10 FRGs that can be used to predict prognosis in patients with oral squamous cell carcinoma. The predictive model of Nie et al. (2021) comprises five FRGs, which can predict the survival rate of patients with colon cancer to a certain extent. There are few studies on the effect of ferroptosis on the occurrence and treatment of OS.

In this study, we developed a novel FRG-based OS prognostic model and investigated its prognostic impact on patients with OS. We also investigated the relationship between these prognostic genes and tumor immunity. This may help to further improve the prognosis of OS.

## 2 Materials and methods

### 2.1 Data sources and processing

We downloaded OS transcriptional data along with the corresponding platform annotation documents and patient clinical profiles from The Cancer Genome Atlas (TCGA) (https://portal.gdc.cancer.gov/repository) and Gene Expression Omnibus (GEO) (https://www.ncbi.nlm.nih.gov/geo). FRGs were derived from the ferroptosis database (http://www.zhounan.org/ferrdb/current/). We used |log2FC|>1 and false discovery rate of <.05 as the threshold to determine the differential expression between OS cell lines and normal samples and drew a Venn diagram to obtain FRGs with different expressions.

### 2.2 Construction of the risk score model

Using the data for patients with OS in TCGA as the training set, the prognosis-related genes were determined *via* LASSO machine learning regression analysis. The risk coefficient (βi) was generated, and the risk assessment model, including the coefficient and gene expression level ( $risk=\sum_{1}^{i}expi*\beta i$ ), was constructed to obtain the risk score of all samples. Using the median risk score as the cut-off point, patients were divided into high- and low-risk groups. Survival curves were used to compare the prognosis of the two groups of patients. The predictive ability of the model was assessed by analyzing the receiver operating characteristic (ROC) curve to obtain the area under the curve. The above analysis was validated using the validation set. Finally, the predictive power of the model was assessed by dividing patients into different subgroups based on their age, sex, and presence of metastases. Patients with OS from the GEO database were used as the validation set to validate the results of the training set.

### 2.3 Nomogram construction and evaluation

Univariate and multivariate COX regression analyses were performed using risk scores and clinical factors (age, sex, and metastasis) to screen for independent prognostic factors. We constructed a nomogram based on the multivariate COX regression results of risk scores. Nomograms were used to predict 3- and 5-year overall survival for each patient. Simultaneously, C-index, calibration curve, and ROC curve were generated to evaluate the prediction power of the model. The above results were used to verify the stability of the results in the validation set.

### 2.4 Gene enrichment analysis

Using the Gene Set Enrichment Analysis (GSEA) database (https://www.gsea-msigdb.org/gsea/msigdb/index.jsp), Gene Ontology and Kyoto Encyclopedia of Genes and Genomes enrichment analyses were performed on high- and low-risk groups, and gene function set analysis was established. The established set was used to determine whether the genomes were statistically significantly different in different biological states.

### 2.5 Correlation analysis of prognostic genes and tumor immunity

CIBERSORT analysis was used to determine the degree of immune cell infiltration in patients with OS. Correlation analysis was then used to determine the association of prognostic FRGs with immune cell infiltration to examine the mechanisms by which prognostic genes influence OS progression.

### 2.6 Cell culture

MG-63 human OS cells were grown in Dulbecco’s modified Eagle’s medium containing 10% fetal bovine serum and 1% penicillin/streptomycin (Gibco; Thermo Fisher Scientific, Waltham, MA, United States) at 37°C and 5% CO_2_. Saos-2 cells were cultured in McCoy’s S5 A medium (37°C, 5% CO_2_) containing 15% fetal bovine serum and 1% penicillin/streptomycin (Gibco). The medium was replaced with a freshly prepared one every other day.

### 2.7 Cell viability assay

MG-63 and Saos-2 cells were seeded onto 96-well plates at a density of 5,000 cells/well and cultured in an incubator at 37°C and 5% CO_2_ for 24 h. Cells were then treated with 0, 10, 20, and 40 µM erastin (Aladdin Biochemical Technology Co., Ltd., Shanghai, China) for 24, 48, and 72 h. Then, Cell Counting Kit-8 (TargetMol) reagent was added, and the 96-well plate was incubated for 2 h. Optical density at 450 nm was measured using a microplate reader. Three independent experiments were performed, each in triplicate.

### 2.8 ROS level measurement

MG-63 and Saos-2 cells were incubated in 60 mm cell culture dishes for 24 h. Then, DMSO (ferroptosis activator erastin, 10, 20, 40 μM) was added to different treatment groups for 48 h, and the samples were incubated for 30 min at 37°C in 60 mm cell culture dishes containing 5 μM BODIPY581/591C11 stain (D3861, Invitrogen, Carlsbad, CA, United States). Cells were washed with PBS, trypsinized, and stained with propidium iodide in PBS for 5 min. They were then stained using the ROS assay kit (Beyotime Institute of Biotechnology, Jiangsu, China) according to the manufacturers’ instructions 23°C ± 2°C for 20 min, and analyzed *via* flow cytometry (CytoFLEX, Beckman, CA, United States) to determine the ROS levels.

### 2.9 Real-time PCR

Total RNA was extracted from Morin-treated MG-63 and Saos-2 cells using the RNeasy Mini Kit (Qiagen, Valencia, CA, United States) according to the manufacturer’s instructions. The concentration and purity of all RNA samples were determined by measuring the absorbance at 260/280 nm. The iScript cDNA Synthesis Kit from Bio-RadTM (Hercules, CA, United States) reversely transcribes 1 µg of RNA. Real-time PCR analysis was performed on an iCycler thermal cycler using the SYBR Green qPCR Supermix kit (Invitrogen, Carlsbad, CA, United States). Real-time quantitative PCR was used to detect the mRNA expression levels of central prognostic genes. Relative mRNA expression of each gene was determined by normalizing it to *GAPDH*. Real-time PCR primers were purchased from Sankong Bio (Shanghai, China) (Supplementary Table S1).

### 2.10 Statistical analysis

All statistical analyses were performed using R 3.3.1 (https://www.r-project.org/). An analytical scoring model was built using LASSO regression. The main packages used were Limma, GSA, GSEABase, Sparcl, Pheatmap, Estimate, ggpubr, e1071, PreprocessCore, Survival, Glmnet, SurvMiner, Survivvalroc, RMS, Foreign, TimeROC, and ggplot2. Differences were considered statistically significant at *p* < .05.

## 3 Results

### 3.1 Differential expression analysis of FRGs

**FIGURE 1:**
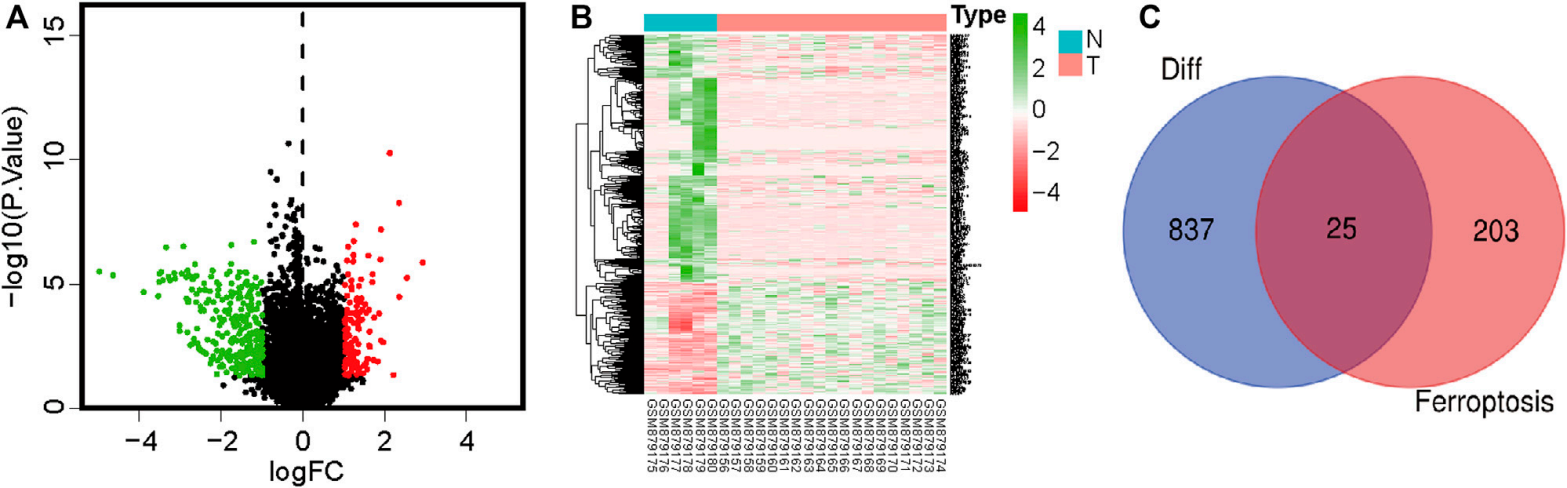
Volcano plot showing differences in gene expression in osteosarcoma and paracancerous tissues. Red color indicates significantly upregulated genes, green color indicates significantly downregulated genes, and black color indicates no significant difference in gene expression. **(A)** Volcano plot showing differentially expressed genes. **(B)** Heatmap of differentially expressed genes. **(C)** Venn diagram showing intersections between differentially expressed genes and ferroptosis-related genes.

We extracted OS cell sample data from the GEO database, including 19 OS cell lines and 6 healthy samples (osteoblasts and bone). Data of 84 and 53 patients with OS with complete clinical data were downloaded from TCGA and GEO, respectively (Supplementary Table S2). FRGs were obtained from the FerrDb database. Differentially expressed genes in OS and normal cells were identified using the Limma R software package. A total of 862 differentially expressed genes were identified, including 596 downregulated and 266 upregulated genes (Figures 1A, B; Supplementary Table S3). Subsequently, 25 differentially expressed FRGs were obtained through the intersection of FRGs (Figure 1C).

### 3.2 Construction of the prognostic risk score model

**FIGURE 2:**
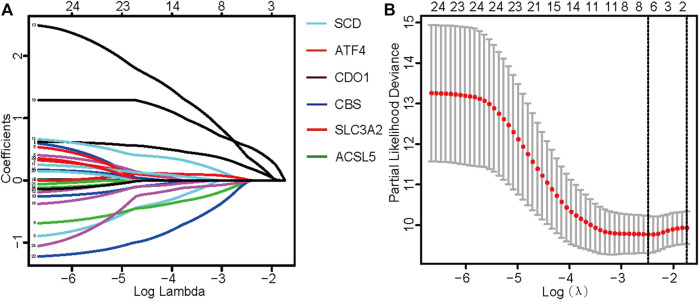
Identification of ferroptosis-related genes with prognostic value for osteosarcoma. **(A)** Fitting parameters of the LASSO regression model. **(B)** LASSO coefficient profile of genes associated with prognosis.

**FIGURE 3:**
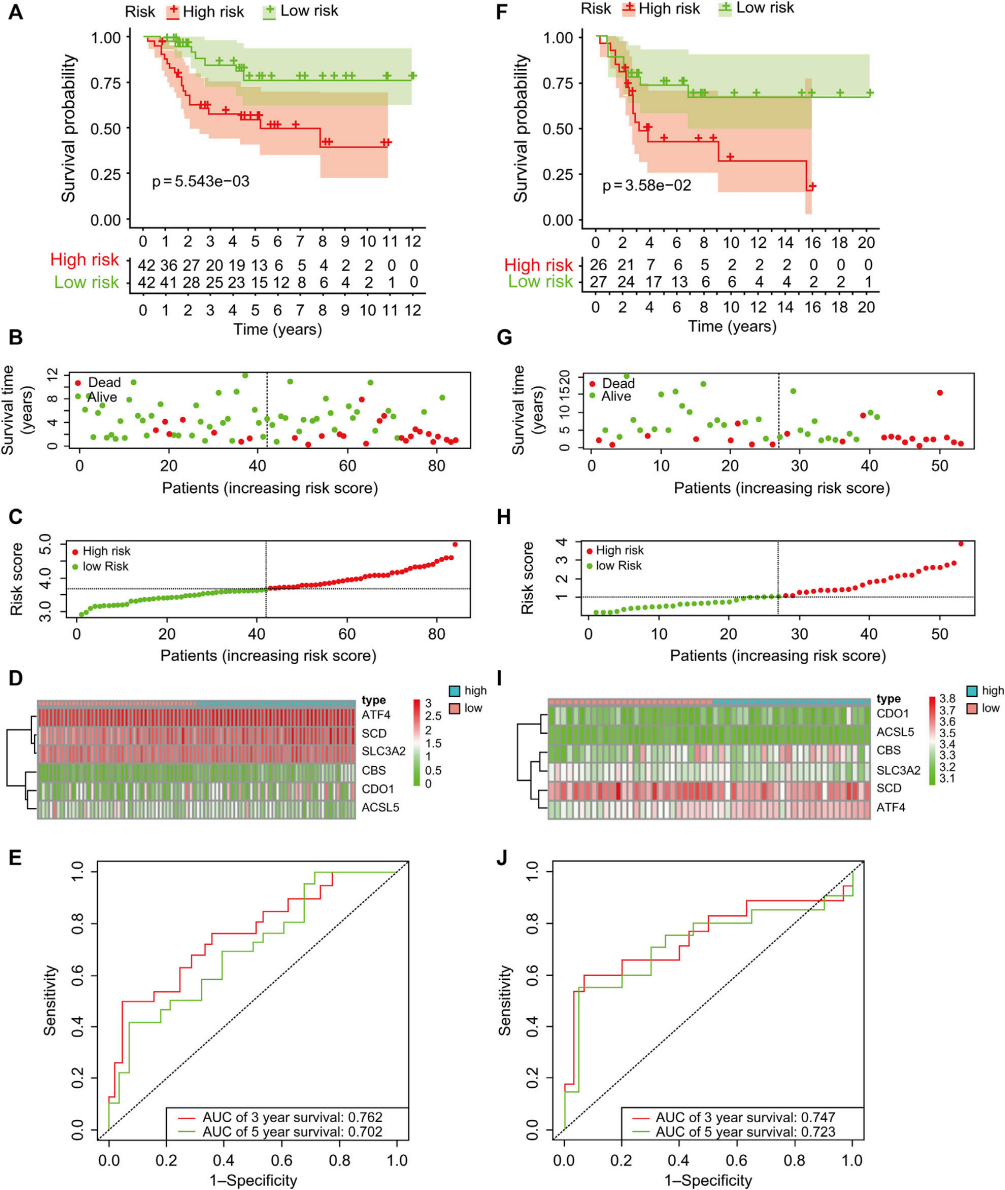
Kaplan–Meier survival analysis of patients with osteosarcoma in the test and validation sets of risk assessment models **(A, F)** showed that the high-risk group showed a significantly poor prognosis, and the low-risk group showed a good prognosis. **(B, G)** Survival of patients with osteosarcoma in the test and validation sets. **(C, H)** Distribution of risk scores for six genes in osteosarcoma patients in test and validation sets. **(D, I)** Heatmaps of six genes in low- and high-risk groups for test and validation sets. **(E, J)** Receiver operating characteristic curve analysis and validation of patients with osteosarcoma in the test set. AUC, area under the curve.

TCGA data were used as the test set, and GEO data were used as the validation set; patients with a follow-up time of more than 30 days were included in the LASSO regression analysis. Six core genes (*ACSL5, ATF4, CBS, CDO1, SCD,* and *SLC3A2*) closely related to prognosis were obtained through screening (Figures 2A, B). A risk scoring model was constructed using multivariate Cox regression analysis (The result was shown in Supplementary Table S4). The median risk score was used as a cut-off point to classify patients into high- and low-risk groups. The survival curve showed that the OS of the low-risk group was significantly higher than that of the high-risk group (*p* < .001, Figure 3A). Risk curves and scatterplots showed that the low-risk group had lower risk and mortality coefficients than the high-risk group (Figures 3A, B). A heatmap showing the expression levels of the six genes in the high-risk and low-risk groups is provided in Figure 3D. The 3- and 5-year area under the curve values obtained from the ROC curves were .762 and .702, respectively (Figure 3E). Similar results were obtained using the GEO data validation set (Figures 3F–J).

### 3.3 Subgroup analysis

**FIGURE 4:**
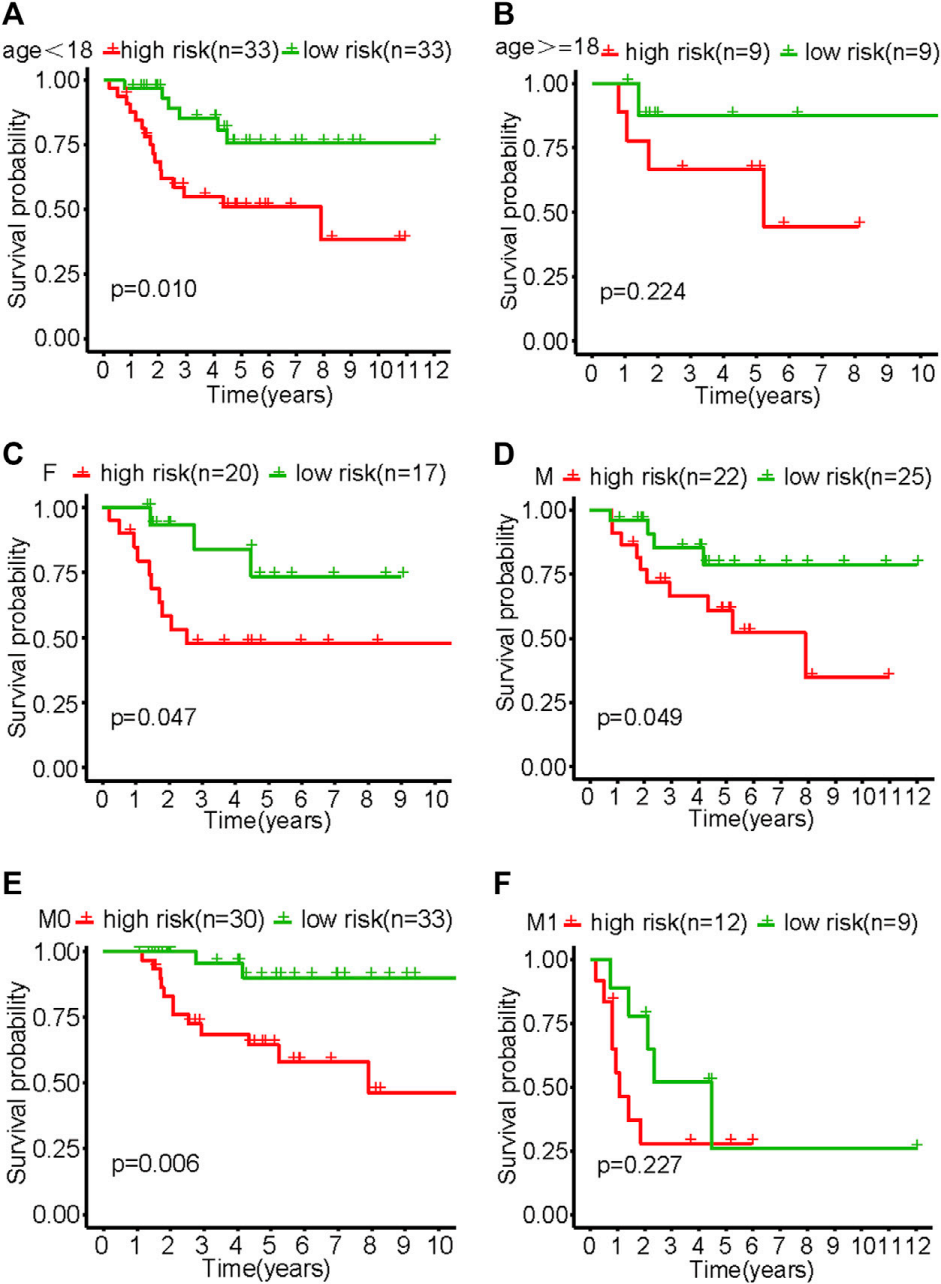
Survival curves of various subgroups in the high- and low-risk groups of patients with osteosarcoma. **(A)** Patients aged <18. **(B)** Patients aged ≥18. **(C)** Females. **(D)** Males. **(E)** M0. **(F)** M1.

The results of the subgroup analysis showed that the model showed better differentiation ability in the subgroups of <18 years old, male, female, and M0 patients (non-metastatic disease) (*p* < .05). Due to the small sample size of patients ≥18 years old and the M1 subgroup (metastatic disease), survival curves tended to differ from baseline but not significantly (Figure 4).

### 3.4 Independent prognostic factor screening

**FIGURE 5:**
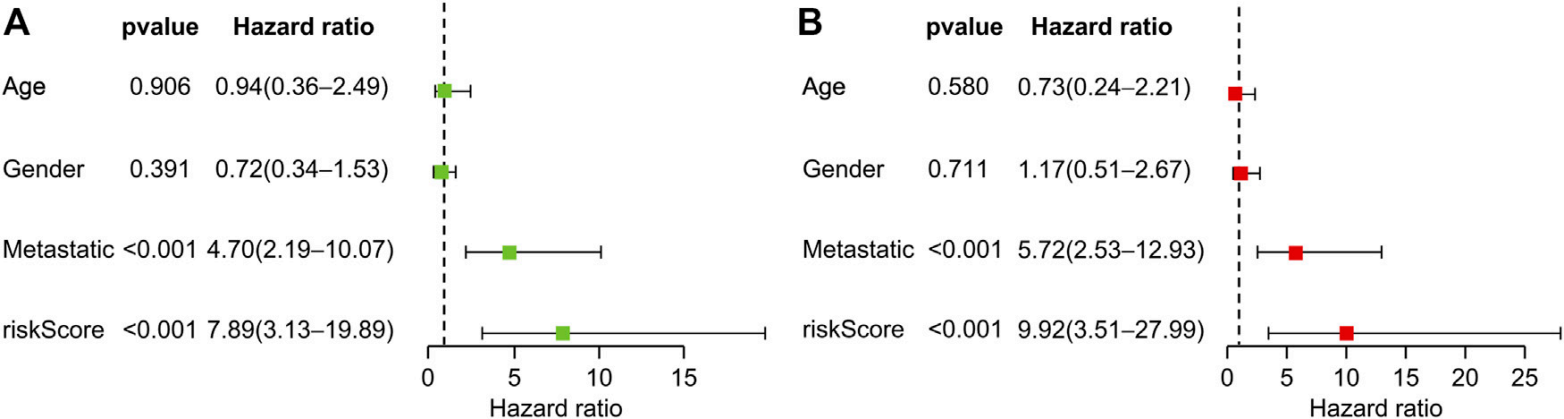
Prognostic value of clinical information and risk score. **(A)** Univariate Cox regression analysis of patients with osteosarcoma. **(B)** Multivariate Cox regression analysis of patients with osteosarcoma.

Univariate and multivariate Cox regression analyses revealed that transfer and risk scores of six genes were independent risk factors for patients with OS (Figures 5A, B). Nomograms were constructed based on independent prognostic factors.

### 3.5 Nomogram construction

**FIGURE 6:**
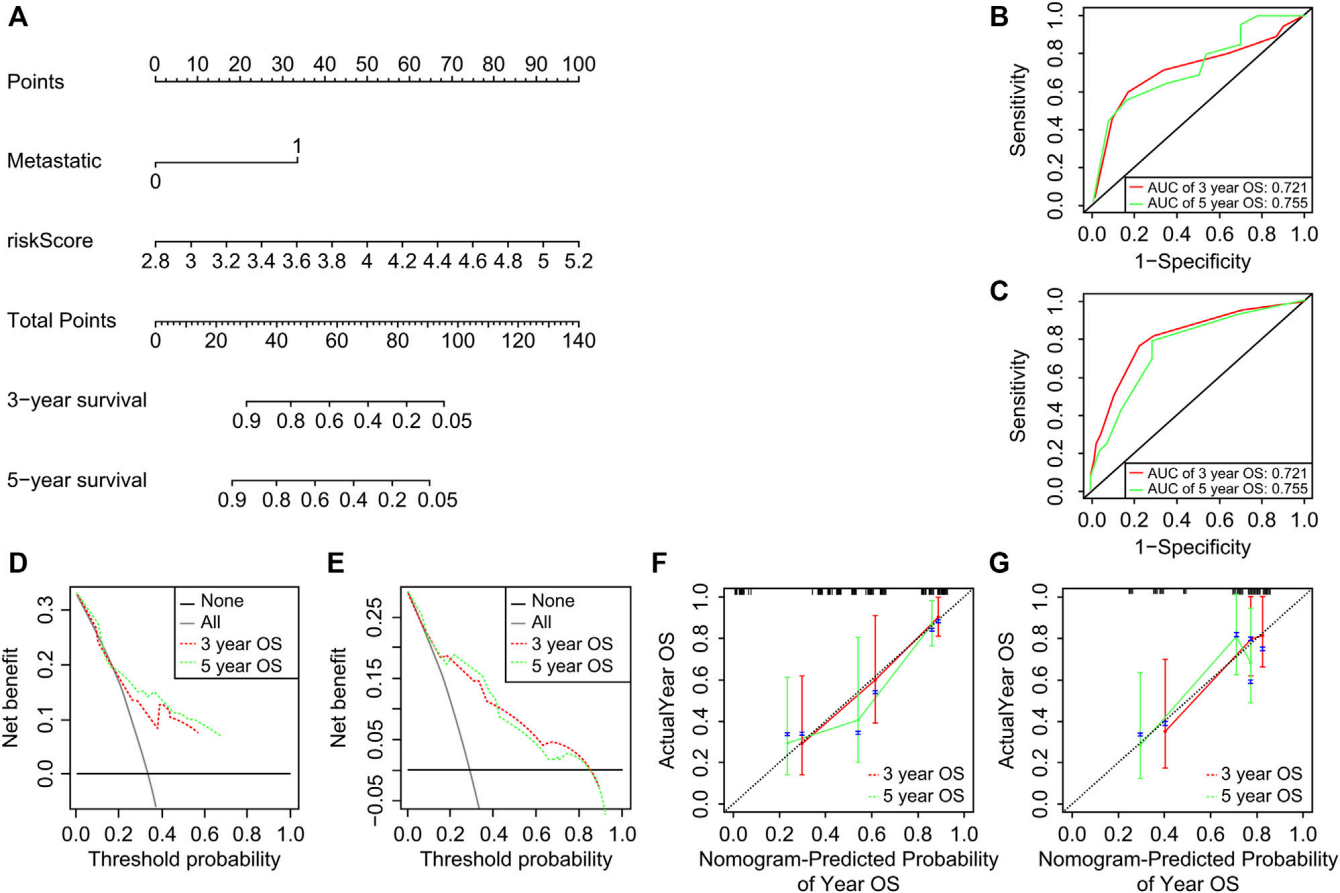
Construction and evaluation of the nomogram. **(A)** Nomogram constructed using the training set. **(B, C)** Receiver operating characteristic curves and validation using the test set. **(D, E)** Decision curve analysis clinical decision curves for the test and validation sets. **(F, G)** Calibration curves for test and validation sets. OS, osteosarcoma.

Results of multivariate Cox regression analysis were used for column plot construction, including both metastasis and risk scores (Figure 6A). Using the C-index of the nomogram as the training set (.804), the area under the curve for overall survival at 3 and 5 years was .741 and .775, respectively (Figure 6B). A clinical decision curve analysis (Figure 6D), and a calibration curve (Figure 6F) were also established. The results showed that the model has good predictive performance and may aid patients. The stability of the above results was validated using the GEO validation set (Figures 6C, E, G).

### 3.6 GSEA

**FIGURE 7:**
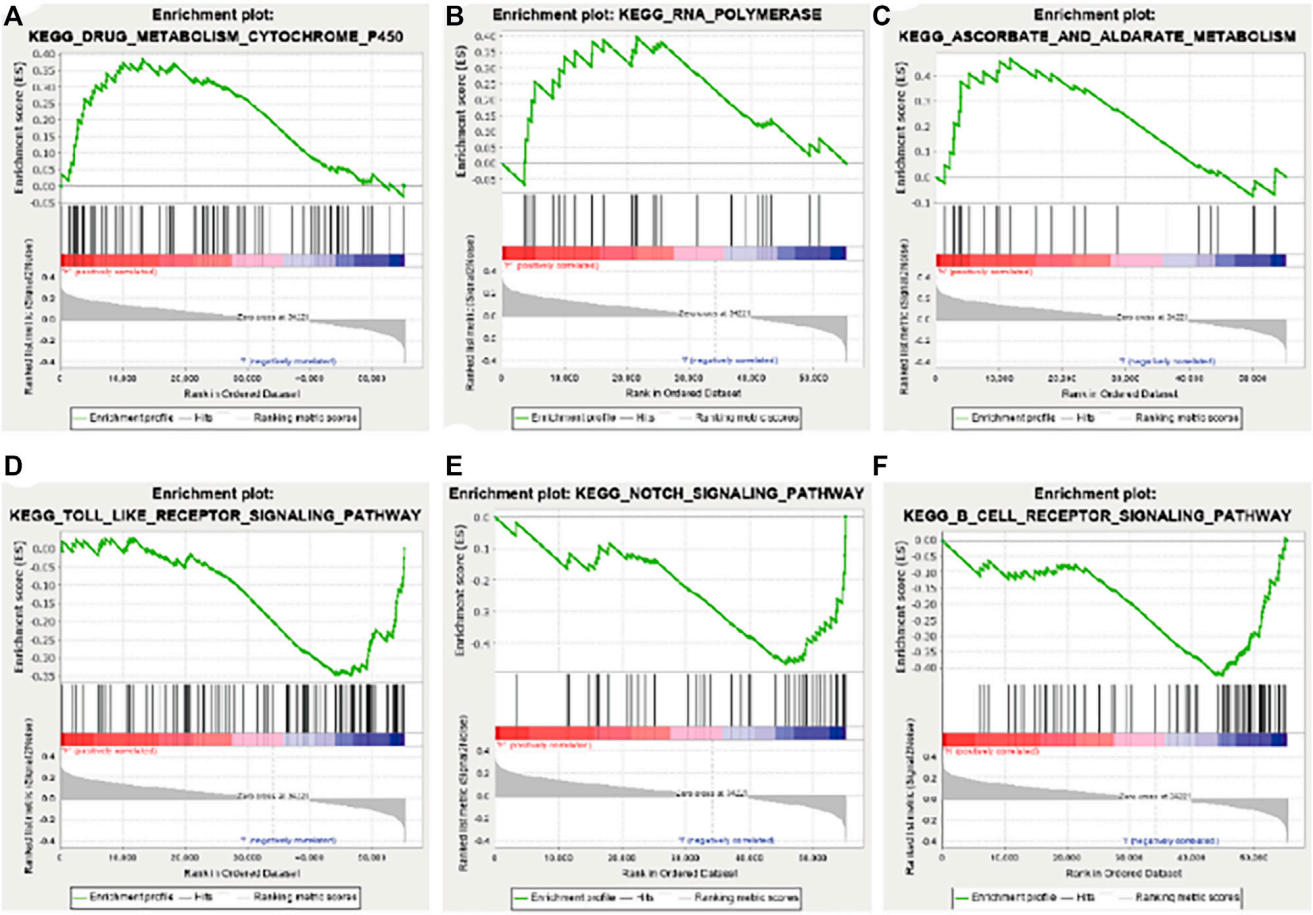
Gene set enrichment analysis enrichment results. **(A)** Drug metabolism cytochrome P450. **(B)** RNA polymerase. **(C)** Ascorbate and aldarate metabolism. **(D)** Toll-like receptor signaling pathway. **(E)** Notch signaling pathway. **(F)** B cell receptor signaling pathway. KEGG, Kyoto Encyclopedia of Genes and Genomes.

GSEA enrichment analysis showed that the activated signaling pathways in the high-risk group were ascorbic acid and uronic acid metabolism, cytochrome P450 drug metabolism, and RNA polymerase (Figures 7A–C), while those in the low-risk group were B cell receptor signaling pathway, Notch signaling pathway, and Toll-like receptor signaling pathway (Figures 7D–F).

### 3.7 Correlation analysis between prognostic genes and tumor immunity

**FIGURE 8:**
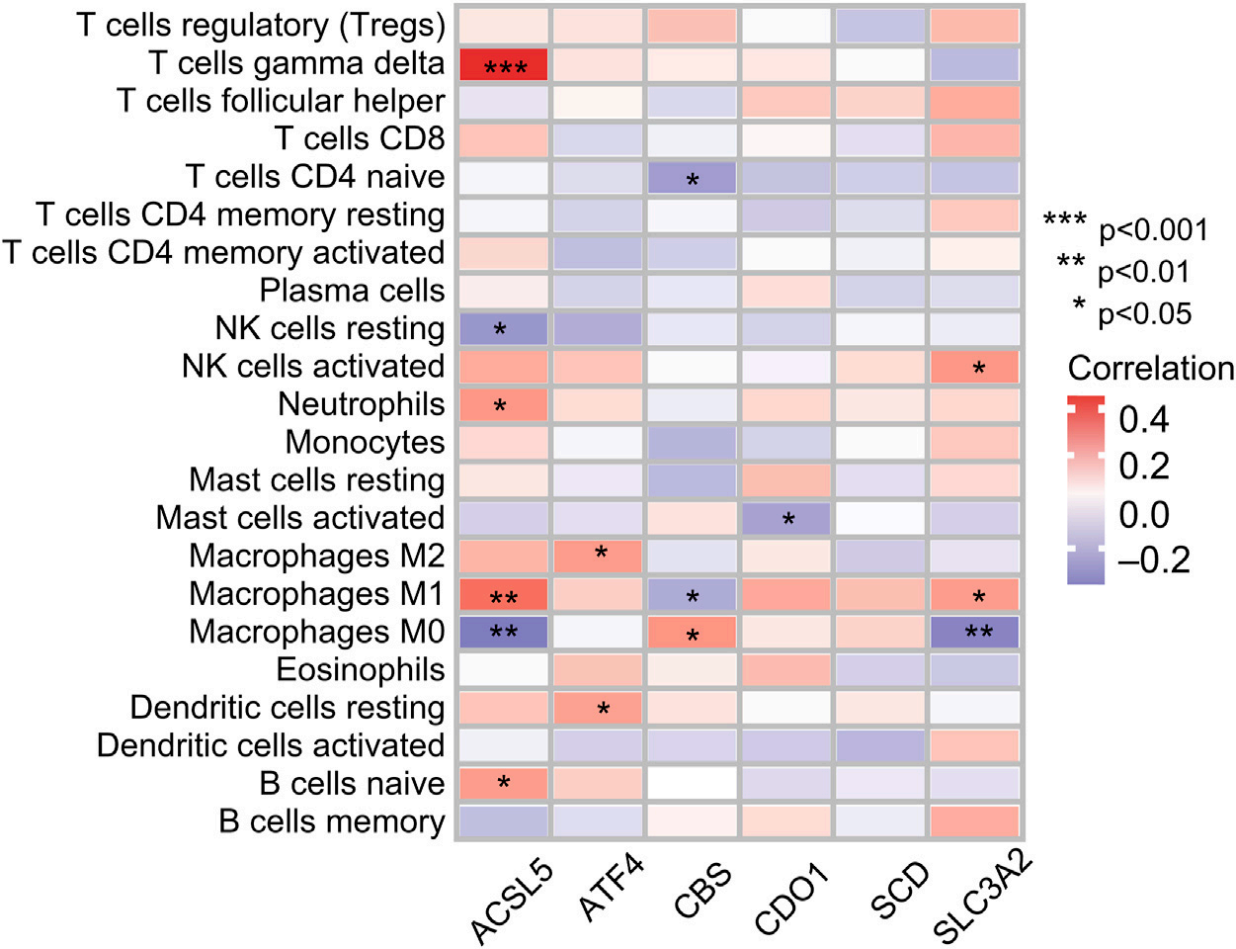
Correlation between immune cell ratios based on CIBERSORT algorithm and expression levels of six genes used for risk score model construction.

Infiltration values of 22 immune cell types in patients with OS were determined using CIBERSORT analysis. The results of the study showed that the genes used for model construction were associated with γ/δ T cells, CD4-naive T cells, NK cell quiescence, NK cells activation, neutrophils, mast cell activation, macrophages, dendritic cell quiescence, and naive B cell infiltration (Figure 8), suggesting that these genes may affect the tumor-infiltrating microenvironment, thereby regulating tumor growth and progression.

### 3.8 Effect of erastin on the expression levels of six genes related to the prognosis of OS

**FIGURE 9:**
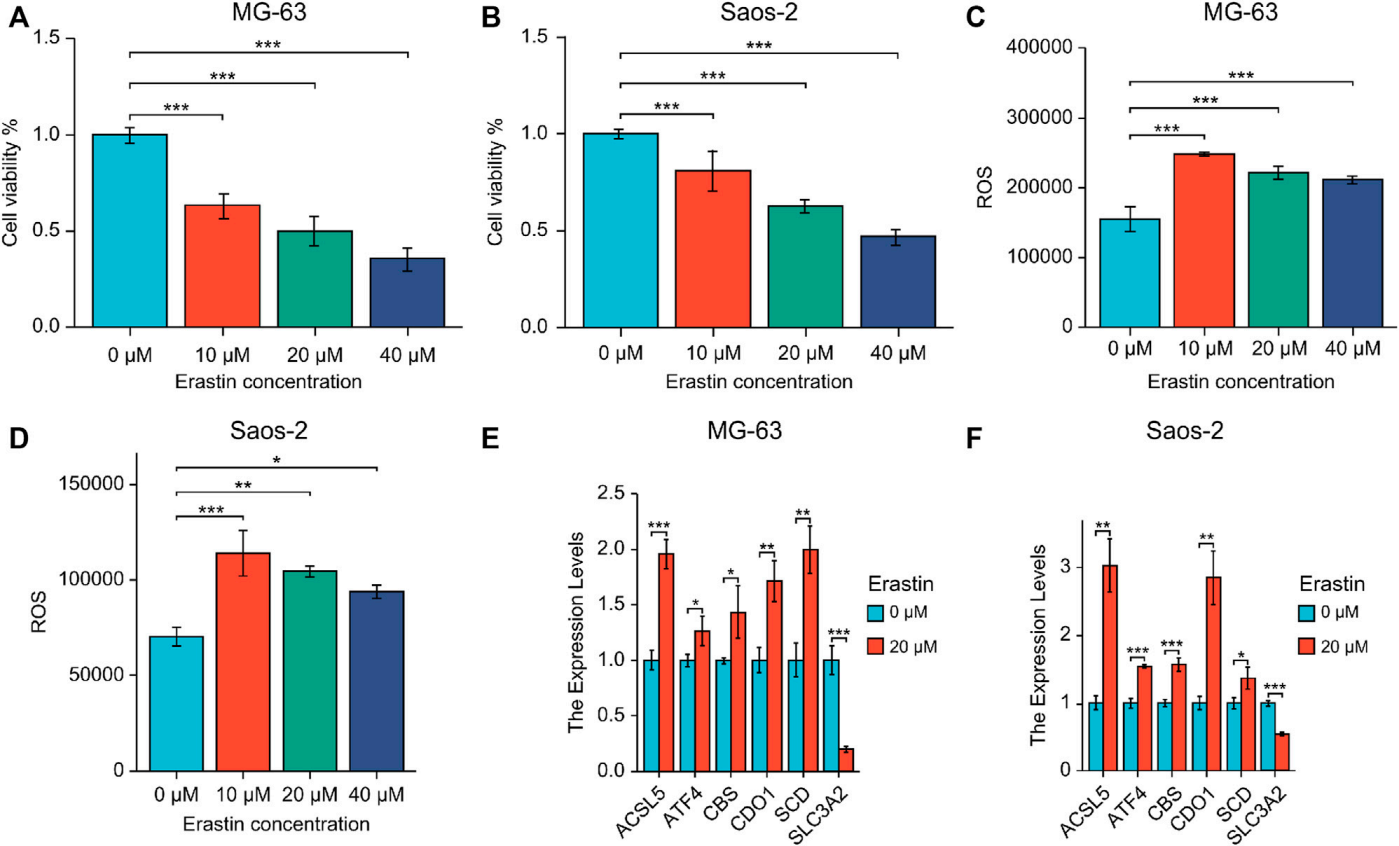
**(A, B)** Expression of six prognostic genes in MG-63 **(A)** and Saos-2 **(B)** cells treated with erastin. **(C, D)** Lipid peroxidation experiments were performed using erastin-treated MG-63 **(C)** and Saos-2 **(D)** cells *via* flow cytometry. **(E, F)** Real-time PCR of six core prognostic genes of MG-63 **(E)** and Saos-2 **(F)** cells upon 20 μM erastin treatment for 48 h. *p < .05; **p < .01; ***p < .001. ROS, reactive oxygen species.

MG-63 and Saos-2-OS cell lines were treated with ferroptosis-triggering erastin to examine the role of six prognostic genes. The results of cell viability assay showed that erastin inhibited the proliferation of MG-63 and Saos-2 cells in a dose-dependent manner (Figures 9A, B). When the concentration of erastin exceeded 10 μM, its growth inhibitory effect on both OS cell lines was significant. We then performed FACS experiments and found that treatment with 10, 20, and 40 μM erastin significantly increased ROS accumulation (Figures 9C, D). Finally, we tested the expression of six nuclear genes in MG-63 and Saos-2 cells treated with 20 μM erastin for 48 h using real-time PCR. As a result, the mRNA expression levels of five genes (*ACSL5, ATF4, CBS, CDO1,* and *SCD*) were elevated after erastin treatment and positively correlated with OS ferroptosis; mRNA expression of *SLC3A2* was decreased and negatively correlated with OS ferroptosis (Figures 9E, F).

## 4 Discussion

OS is a common malignancy that threatens adolescent health globally, and its incidence and mortality rate increase every year. Previous studies have shown that ferroptosis is strongly related to the prognosis of OS. Screening for reliable ferroptosis-related prognostic markers can improve the prognosis of patients with OS and help provide reliable information to guide OS treatment (Zhao et al., 2021).

In this study, we constructed an OS prognostic model using FRGs to predict the survival of patients with OS. This model consisted of six genes. At the same time, it showed good prediction performance and could benefit patients with OS. We further analyzed the correlation between prognostic genes and tumor immunity. Finally, we tested the effects of ferroptosis-inducing factors on the expression of six prognostic genes in OS cell lines *in vitro*.

Ferroptosis is an iron-dependent and non-apoptotic cell death mechanism (Shimada et al., 2016; Du et al., 2020). In tumor cells, induction of ferroptosis improves treatment, making it a current research hotspot (Chen et al., 2020; Jin et al., 2021). Ferroptosis plays an important role in OS treatment, particularly in drug-resistant OS cells (Qiu et al., 2022). However, there are few studies on whether specific FRGs regulate OS progression at present. The link between ferroptosis and the prognosis of OS has not been demonstrated. Our study showed that 25 FRGs were significantly differentially expressed in OS and normal cells, suggesting that FRGs may be involved in ferroptosis and that it is possible to use FRGs to build predictive models.

The six FRGs used for model construction were *ACSL5, ATF4, CBS, CDO1, SCD,* and *SLC3A2*. ACSL5 is a mitochondrial enzyme that can promote the synthesis of long-chain fatty acid thioesters and can promote apoptosis. ACSL5 isoenzymes play a dominant role in cardiolipin biosynthesis in mitochondria and may participate in cancer cell survival (Zhang et al., 2019). Previous research has shown that ACSL5 may be vital to the malignant progression and metastasis of gliomas, and this supports targeting ACSL5 as a potentially effective treatment strategy (Sebastiano and Konstantinidou, 2019). ATF4 is a transcription factor that is associated with the progression of different cancers, such as breast cancer, lung cancer, and melanoma. ATF4 is highly expressed in human OS, and MYC-regulated ATF4 may help in anoikis resistance in human OS cells (Mo et al., 2018). CBS is one of the three main enzymes that participate in H_2_S biosynthesis in various mammalian cells and tissues (Russo et al., 2016). Studies reported that CBS is highly expressed in colon cancer (Szabo et al., 2013), ovarian cancer (Bhattacharyya et al., 2013), prostate cancer (Guo et al., 2012), and breast cancer (Sen et al., 2015). Pharmacological inhibitors or silencing of CBS were associated with anticancer effects *in vitro* and *in vivo*, increasing the effectiveness of L-OHP (Russo et al., 2016). CDO1 is a tumor suppressor in human cancers and can promote apoptosis. As *CDO* is a methylation-specific gene in human cancers, CDO1 methylation has been reported in many cancers recently (Nishizawa et al., 2019; Alfarsi et al., 2020). SCD is a rate-limiting enzyme in lipid biosynthesis that plays a key role in fuel metabolism and is a potential therapeutic target in cancer treatment (Piao et al., 2019; Zhou et al., 2021). Overexpression of SLC3A2 can promote tumorigenesis, and SLC3A2 is overexpressed in various cancer cell lines including lung cancer, colon cancer, and breast cancer. SLC3A2 is also known as a new marker for kidney cancer (Nguyen et al., 2018).

The interaction between tumor cells and the immune microenvironment plays an important role in the occurrence and development of tumors (Kim et al., 2021). The tumor microenvironment consists of cellular components, including immune cells, endothelial cells, fibroblasts, and non-cellular components, including the extracellular matrix, cytokines, and hormones. Immune cells play an important role in influencing tumor behavior and its response to treatment (Wu and Dai, 2017). In our study, we performed a correlation analysis of tumor immunity based on ferroptosis-related prognostic genes. Interestingly, we found that the FRGs used in model construction were correlated with the infiltration of some immune cells. Wang W. et al. (2019) were the first to reveal that immunotherapy-activated CD8^+^ T cells induce ferroptosis in ovarian tumor cells, showing that ferroptosis is intimately associated with antitumor immunity. Furthermore, immunotherapy-activated CD8^+^ T cells induce ferroptosis in human melanoma and fibrosarcoma cells (Lang et al., 2019). Another study reported the identification of a new gene feature about ferroptosis, prognosis prediction, and immune microenvironment of OS and suggested that FRGs in patients with OS were closely related to the immune microenvironment (Zheng et al., 2022).

γδ T cells show varying degrees of cytolytic activity toward different malignant tumors, and *in vitro* expansion of γδ T cells may be a promising immunotherapy option against malignant tumors (Wu et al., 2014; Long, 2018). We found that the FRG ACSL5 might be intimately associated with the immune responses of γ/δ T cells in OS. Although the specific mechanisms behind this require further clarification, this may help to provide new insights into the molecular mechanisms underlying the origin and development of OS, as well as to explore potential targeted therapies for OS, which would have significant clinical implications, especially for patients with poor prognosis.

There are some limitations to this study. First, we constructed and validated a prognostic model related to ferroptosis based on a public database of retrospective data. We also demonstrated that ferroptosis-inducing erastin affects the expression of six important prognostic genes in OS cells *in vitro*. In the future, we will use multicenter prospective clinical data to further confirm the above results. In addition, the correlation between our model and tumor immunity requires further experiments for validation.

In conclusion, we constructed an FRG-based OS prognostic model that has good prediction performance. We also showed that FRGs are significantly correlated with the invasion of immune cells. This study can provide a reference for the development of new treatments for OS.

## Data Availability

The original contributions presented in the study are included in the article/Supplementary Material, further inquiries can be directed to the corresponding authors.

## Abbreviations

OS, osteosarcoma; FRG, ferroptosis-related gene; TCGA, The Cancer Genome Atlas; GEO, Gene Expression Omnibus; ROS, reactive oxygen species; ROC, receiver operating characteristic; GSEA, Gene Set Enrichment Analysis.
