# Supplementary material for: Employing machine learning using ferroptosis-related genes to construct a prognosis model for patients with osteosarcoma
Source: Front Genet. 2023 Jan 17;14:1099272. doi: 10.3389/fgene.2023.1099272 (PMC9888665; doi:10.3389/fgene.2023.1099272)
Supplement: Supplementary file 2 [file Table2.DOCX]

Supplementary Material

Employing machine learning using ferroptosis-related genes to construct a prognosis model for patients with osteosarcoma

Hui Huang^1^, Zhifang Ye^1^, Zhengzhao Li^2^, Bo Wang^1^, Ke Li^1^, Kai Zhou^1^, Huiyuan Cao^1^, Jiaxuan Zheng^3^ and Guangji Wang^1^

*** Correspondence:** Guangji Wang: hhall517@163.com; Jiaxuan Zheng: 342979919@qq.com

# Supplementary Figures and Tables

## Supplementary Tables

**Supplementary Table 1. Primers used in qRT-PCR**

**Supplementary Table 2. Baseline patient data in training and validation sets**

| Item | Training cohort  (TCGA n = 84) | Validation cohort  (GSE21257 n = 53) |
| --- | --- | --- |
| Age, n (%) |  |  |
| ≥ 18 | 18(22.1) | 20(37.7) |
| < 18 | 66(77.9) | 33(62.3) |
| Gender, n (%) |  |  |
| Female | 37(40.7) | 19(35.8) |
| Male | 47(59.3) | 34(64.2) |
| Metastasis, n (%) |  |  |
| Non-metastatic | 63(74.4) | 19(35.8) |
| Metastasis | 21(25.6) | 34(64.2) |

**Supplementary Table 3. Differentially expressed genes in osteosarcoma and normal cells**

**Supplementary Table 4. The results of cox regression analysis**
